# Comment on Mureșan et al. Prognostic Nutritional Index, Controlling Nutritional Status (CONUT) Score, and Inflammatory Biomarkers as Predictors of Deep Vein Thrombosis, Acute Pulmonary Embolism, and Mortality in COVID-19 Patients. *Diagnostics* 2022, *12*, 2757

**DOI:** 10.3390/diagnostics15172151

**Published:** 2025-08-26

**Authors:** Mengyi Lian, Chengwei Zhang

**Affiliations:** 1 Department of Obstetrics and Gynecology, Longquan People’s Hospital, Lishui 323700, China; mengyilian0078@gmail.com; 2 Innovative Regenerative Medicine, Graduate School of Medicine, Kansai Medical University, Osaka 573-1191, Japan

**Keywords:** COVID-19, deep vein thrombosis, pulmonary embolism, prognostic biomarkers, multivariate analysis

## Abstract

We read with interest the study by Mureșan et al. on nutritional and inflammatory biomarkers predicting thromboembolic events and mortality in COVID-19 patients. The simultaneous independent significance of eight correlated biomarkers raises concerns about possible multicollinearity. We welcome the authors’ clarification and updated multivariate results, which confirmed that several markers lost significance after adjustment. We recommend clearer statistical terminology and parallel reporting of univariate and multivariate results to improve transparency.

With great interest, we read the article by Manole et al. on inflammatory biomarkers as prognostic factors of acute deep vein thrombosis (DVT) following total knee arthroplasty. We wish to present our comments on the method for multivariate analysis for predictors of DVT, acute pulmonary embolism (APE), and mortality in COVID-19 patients.

In November 2022, the manuscript by Adrian Vasile Mureșan et al. was published in *Diagnostics* [1]. The authors confirmed that the prognostic nutritional index (PNI), CONUT Score, and inflammatory markers (monocyte-to-lymphocyte ratio (MLR), neutrophil-to-lymphocyte ratio (NLR), platelet-to-lymphocyte ratio (PLR), systemic inflammatory index (SII), systemic inflammation response index (SIRI), and aggregate index of systemic inflammation (AISI)) have predictive value for the risk of DVT, APE, and mortality in COVID-19 patients.

In their results (page 6), the authors stated: “A multivariate analysis was used to determine the association between all markers, underlying risk factors, DVT, APE development risk, and mortality during the hospitalization. A high baseline value of all systemic inflammatory markers and CONUT Score was a strong independent predictor of all outcomes (for all *p* < 0.0001)”. We consider it methodologically implausible that all eight indicators would simultaneously remain independent risk factors. As we are engaged in related research, we note that these hematological indicators are often closely correlated, with potential collinearity. Therefore, it is statistically unlikely for all of them to remain significant after multivariate adjustment. Several inflammatory indices for predicting DVT have been evaluated in other studies, showing strong predictive effects in univariate logistic regression analysis; however, because these indicators are not fully independent, typically only one or two remain as independent risk factors in multivariate models [2,3,4].

We also examined the statistical methods used in the study. The authors did not describe in detail how the multivariate logistic regression analysis was performed, nor did they provide separate odds ratios (ORs) for univariate and multivariate analyses, as is standard in similar research [3,5]. Based on the authors’ univariate analysis results, we calculated the OR for high-PLR patients: (139/169)/(52/529) = 8.36, which matched the OR reported in the multivariate analysis. Normally, when conducting multivariate analysis, the OR will differ from the univariate result due to adjustment for covariates [6]. This raises the possibility that univariate and multivariate results may have been conflated. We would appreciate it if the authors could provide separate results for univariate and multivariate logistic regression analyses, or present correlation analyses among the various indicators to demonstrate that they are not substantially interdependent.

After raising our concerns, we received a response from the authors. We appreciate their clarification that the term “multivariate analysis” was mistakenly used and that the results originally presented in Table 1 were from univariate analyses. Furthermore, the authors have now provided multivariate regression results with three adjustment models. Their updated analysis confirms our initial concern: when adjusting for comorbidities and other biomarkers, several indices (including SII, AISI, and PNI) lost statistical significance for predicting DVT; MLR, PLR, SII, SIRI, and PNI for APE; and MLR, SIRI, and AISI for mortality.

We commend the authors for acknowledging the oversight and for providing corrected statistical analyses. Their updated findings not only support the validity of their overall conclusions but also provide a more precise interpretation of the independent predictive value of each biomarker. We recommend that the journal editorial team consider issuing a corrigendum to rectify the analytical terminology and improve clarity for future readers.

This case underscores the importance of clearly distinguishing between univariate and multivariate results and the need to report odds ratios (ORs), confidence intervals (CIs), and model covariates for both types of analysis. We appreciate the authors’ transparency and their contribution to improving scientific rigor.
